# Hyperacute transplantation of umbilical cord mesenchymal stromal cells in a model of severe intracerebral hemorrhage

**DOI:** 10.2144/fsoa-2021-0121

**Published:** 2022-03-24

**Authors:** Tanira Giara Mello, Paulo Henrique Rosado-de-Castro, Juliana Ferreira Vasques, Carolina Pinhão, Tayná Monteiro Santos, Renata Rodrigues de Lima, Bernd Uwe Foerster, Fernando Fernandes Paiva, Rosalia Mendez-Otero, Pedro Moreno Pimentel-Coelho

**Affiliations:** 1 Instituto de Biofísica Carlos Chagas Filho, Universidade Federal do Rio de Janeiro, Rio de Janeiro, RJ, 21941-902, Brazil; 2 Instituto de Engenharia Nuclear, Comissão Nacional de Energia Nuclear, Rio de Janeiro, RJ, 21941-614, Brazil; 3 Instituto Nacional de Ciência e Tecnologia em Medicina Regenerativa, Rio de Janeiro, RJ, 21941-902, Brazil; 4 Departamento de Radiologia, Faculdade de Medicina, Universidade Federal do Rio de Janeiro, Rio de Janeiro, RJ, 21941-902, Brazil; 5 Instituto de Ciências Biomédicas, Universidade Federal do Rio de Janeiro, Rio de Janeiro, RJ, 21941-902, Brazil; 6 Instituto de Física de São Carlos, Universidade de São Paulo, São Carlos, SP, 13566-590, Brazil

**Keywords:** cell therapy, hemorrhagic stroke, mesenchymal stromal cells, stem cells, Wharton’s jelly

## Abstract

**Aim:**

Intracerebral hemorrhage (ICH) has limited therapeutic options. We have shown that an intravenous injection of human umbilical cord-derived mesenchymal stromal cells (hUC-MSC) 24 h after an ICH in rats reduced the residual hematoma volume after a moderate hemorrhage but was inefficient in severe ICH. Here, we investigated whether a treatment in the hyperacute phase would be more effective in severe ICH.

**Materials & methods:**

Wistar rats were randomly selected to receive an intravenous injection of hUC-MSC or the vehicle 1 h after a severe ICH.

**Results:**

The hyperacute treatment with hUC-MSC did not affect the 22-day survival rate, the motor function or the residual hematoma volume.

**Conclusion:**

These results indicate the need for optimization of hUC-MSC-based therapies for severe ICH.

## Plain language summary

Hemorrhagic stroke, caused by the leakage of blood from blood vessels to the brain, is a life-threatening condition that reduces the quality of life of a large number of patients worldwide without effective treatments. Here, we induced a severe hemorrhagic stroke in rats to study the effects of a treatment using mesenchymal stromal cells, stem cells obtained from the umbilical cord tissue capable of producing protective molecules for the brain. The treatment; however, did not improve some aspects of the disease, such as the motor ability and the size of the brain lesion, indicating that further studies are still necessary.

Intracerebral hemorrhage (ICH) is a common cause of neurological morbidity in adults, corresponding to around 26% of the cases of stroke [1]. Despite the advances in the management of stroke patients, ICH still has high mortality and morbidity rates, which reflects the lack of treatments capable of preventing hematoma expansion and protecting neural cells [2–4].

Mesenchymal stromal cells (MSC) are multipotent cells that can be obtained from a variety of tissues and which can be induced to differentiate into cells of the mesodermal lineage (adipocytes, chondrocytes and osteocytes). MSC constitutively secrete several trophic factors and inflammatory mediators, as well extracellular vesicles carrying biologically active molecules, which have been shown to have neuroprotective, antioxidant and immunomodulatory properties [5]. Moreover, the intravenous (iv.) injection of allogeneic MSC has been shown to be well tolerated in humans [6]. This has opened new possibilities for the utilization of MHowever, whereasSC-based therapies, which have emerged as promising candidates for several central nervous system (CNS) disorders, including stroke [7,8]. However, whereas there are robust preclinical evidence showing the efficacy of the iv. transplantation of MSC in ischemic stroke models [9], there are much less data in experimental ICH [7,10]. We recently have shown that the iv. infusion of human umbilical cord Wharton’s jelly-derived mesenchymal stromal cells (hUC-MSC) 24 h after the hemorrhagic insult can decrease the residual hematoma volume in rats with moderate ICH, but not in animals with severe ICH [11]. This led us to ask whether better outcomes could be obtained in severe ICH if hUC-MSC were injected earlier. This hypothesis was based on data from two meta-analyzes showing greater effect sizes of MSC treatments given within the first 8 h after the insult in experimental ischemic stroke [9,12]. Moreover, ICH is a medical emergency and MSC have the potential to target early pathophysiological processes that are initiated in the first hours after ICH, including oxidative stress, the activation of microglia, the influx of immune cells and the production of pro-inflammatory mediators [5,13]. The aim of this study was to investigate the therapeutic potential of hUC-MSC iv. transplanted 1 h after the induction of a severe ICH in rats.

## Materials & methods

### Animals

A total of 34 male Wistar rats aged 7–10 weeks (mean age: 57 ± 4 days) and weighing 244–305 g were used in this study. All procedures were approved and conducted in accordance with the Animal Care and Use Committee at the Universidade Federal do Rio de Janeiro (protocol number 111/14), and in compliance with the ARRIVE guidelines. All animals received humane care in compliance with the ‘Principles of Laboratory Animal Care’ formulated by the National Society for Medical Research and the US National Academy of Sciences Guide for the Care and Use of Laboratory Animals.

### ICH model

To model a severe ICH, 34 rats received an intrastriatal injection of 0.25 U collagenase IV-S (Sigma-Aldrich, MO, USA) in the left striatum, as previously reported [11]. Animals were treated with tramadol (12.5 mg/kg) as a preemptive analgesic to decrease postoperative pain 15 min prior to the induction of anesthesia with an intraperitoneal injection of xylazine hydrochloride (15 mg/kg) and ketamine hydrochloride (100 mg/kg). After local scalp anesthesia with an intradermal injection of lidocaine (5 mg/kg), an incision was made in the scalp and a hole approximately 1.5 mm in diameter was drilled in the skull to allow the introduction of a 26-gauge needle attached to a 10 μl syringe (Hamilton, NV, USA) [11]. We then injected 2 μl of sterile saline containing 0.25 U of bacterial collagenase at a constant rate of 0.25 μl/min using the following stereotactic coordinates: 3 mm lateral to midline, 0.2 mm posterior to bregma, 6 mm below the surface of the skull. After infusion, the needle was left in place for 10 min, and then withdrawn slowly to prevent backflow. The burr hole was sealed with dental cement, the skin was sutured, and rats were placed in clean cages with free access to food and water. Normothermia was maintained during and after surgery using a heating pad and heating lamps [11].

### hUC-MSC isolation & culture

Umbilical cords were collected from term deliveries after the informed consent forms were signed by the mothers [11]. Umbilical cords were cut into smaller pieces, and dissected for the removal of the arteries and the vein, leaving only the Wharton’s jelly as previously described [11]. The remaining tissue was then digested with collagenase II (200 U/ml; Gibco) diluted in phosphate-buffered saline (PBS) for 16 h at 37°C under slow stirring. The digested material was washed in PBS and plated in Dulbecco’s Modified Eagle’s Medium: Nutrient Mixture F-12 (DMEM/F12; Invitrogen) culture medium supplemented with 15% fetal bovine serum; Invitrogen and 1% penicillin/streptomycin (Gibco, CA, USA). Cells were kept at 37°C in an incubator with 5% CO_2_. After reaching confluence, cells were enzymatically dissociated by incubation with a trypsin solution (0.25% trypsin + 1 mM ethylenediaminetetraacetic acid; Gibco) for 5 min [11]. For cryopreservation, cells at passage number 3–5 were resuspended in fetal bovine serum containing 10% dimethyl sulfoxide (Sigma-Aldrich), and frozen in liquid nitrogen [11]. We used hUC-MSC from a single donor to reduce donor-related variability, and the confirmation of MSC identity has been previously described [14]. Briefly, hUC-MSC were immunophenotyped by flow cytometry, confirming the presence of the cell surface markers CD90, CD73 and CD105, and the lack of CD45 and HLA-DR expression [14].

### hUC-MSC administration

**Figure 1.**
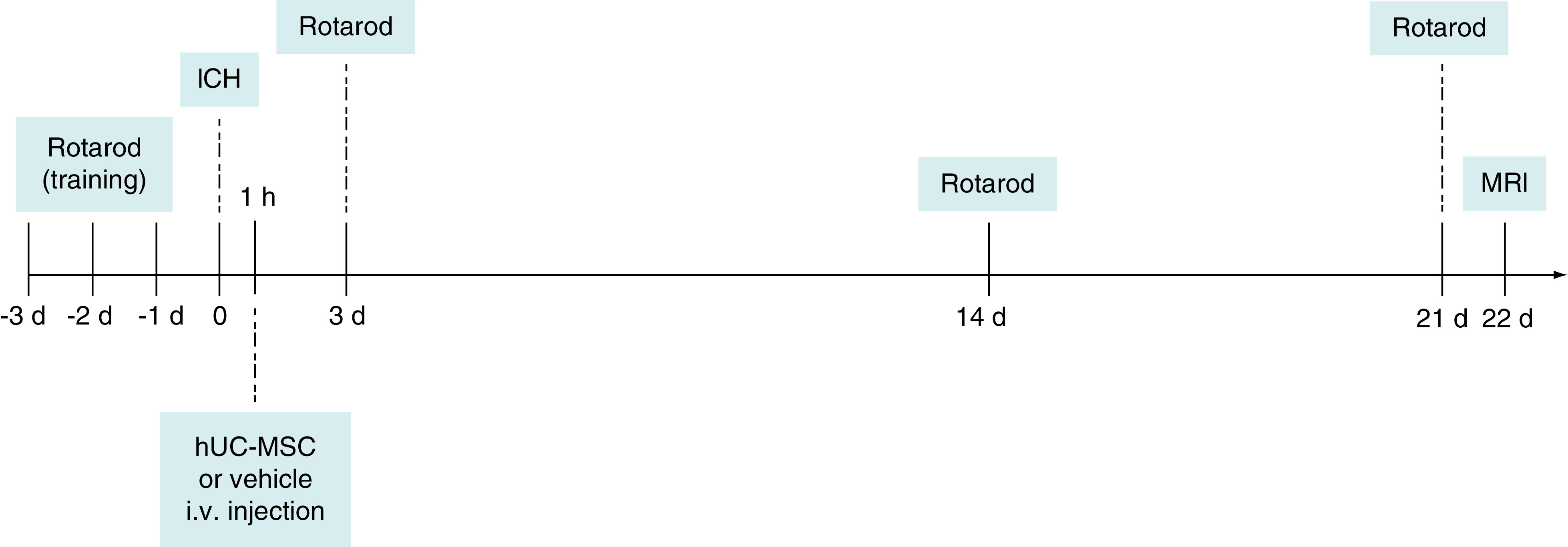
Experimental design. hUC-MSC: Human umbilical cord-derived mesenchymal stromal cells; ICH: Intracerebral hemorrhage; iv.: Intravenous; MRI: Magnetic resonance imaging.

Three animals died within the 1st h following ICH and the remaining 31 animals were randomly selected to receive either a single iv. injection of 3  ×  10^6^ hUC-MSC (ICH + hUC-MSC group; n = 16) or the vehicle (ICH + vehicle group; n = 15) via the tail vein, infused over 2–3 min, 1 h after ICH (Figure 1). Cryogenic vials containing hUC-MSC were defrosted, cells were then centrifuged at 300 × g for 5 min, washed three-times with PBS containing Pulmozyme (recombinant human DNase I; 0.6 μl/ml; Roche) and resuspended in 0.5 ml of PBS + Pulmozyme. The vehicle injection consisted of 0.5 ml of PBS + Pulmozyme [11].

### Rotarod

The rotarod test was employed to assess motor coordination and balance. Animals were pretested by an examiner blinded to treatment allocation for three consecutive days before ICH induction, and then tested on days 3, 14 and 21 after ICH (Figure 1). Baseline assessment after ICH induction was not performed due to the early treatment paradigm. The animals were placed in a neutral position and the rod was set to accelerate from 8–37 rp. in 320 s (Insight Equipamentos, Brazil) [11]. Rats were subjected to three trials per session, with an interval of 5 min between each trial. The time spent on the rotarod (i.e., the latency to fall) was recorded in each trial. For statistical analysis, the best trial (i.e., the longest time spent on the rotarod) was chosen for each animal [11].

### Magnetic resonance imaging

MRI was used to measure the residual hematoma volume (Figure 1) as previously reported [11]. On day 22 after ICH, rats were deeply anesthetized via an intraperitoneal injection of a mixture of xylazine hydrochloride (15 mg/kg) and ketamine hydrochloride (100 mg/kg) and then transcardially perfused with ice cold 0.9% saline, followed by 4% paraformaldehyde in phosphate buffer, pH 7.4. The heads were kept in 4% paraformaldehyde until image acquisition.

MRI was carried out in a 2.0 Tesla Magnetic Resonance System composed of an Oxford Instruments 85310HR Magnet (Oxford Instruments, Abingdon, UK) and (Bruker Avance, Ettlingen, Germany) AVIII console (Bruker-Biospin) at the Instituto de Física de São Carlos, Universidade de São Paulo. A locally developed solenoid coil was used as a transmission and reception coil. T1-, T2- and T2*-weighted 3D sequences were acquired using the same geometric parameters: field of view (FOV) = 32 × 32 × 32 mm with a matrix of 128 × 128 × 128, resulting in an isotropic spatial resolution of 250 μm. The images were reconstructed using zero filling for a 256 × 256 × 256 matrix. T1-weighted images were acquired with a gradient echo sequence with the following parameters: TE/TR = 15/3.5 ms, flip angle = 30 degrees, bandwidth = 15 kHz. T2-weighted images were acquired using ac sequence with the following parameters: TR/TE: 24/7 ms, flip angle = 60 degrees, bandwidth = 15 kHz. Finally, T2*-weighted images were acquired with gradient echo sequence with the following parameters: TE/TR = 20/10.5 ms, flip angle = 5 degrees, bandwidth = 15 kHz. The residual hematoma volume was calculated using the medical image processing, analysis and visualization application ([MIPAV]: 8.0.2; NIH) by an examiner blinded to treatment allocation. To evaluate the residual hematoma volume, the region of interest was marked around the residual hematoma in all coronal sections where it was visible [11]. One animal from the ICH + vehicle group was excluded from this analysis due to the appearance of technical artifacts in the MRI images.

### Statistical analysis

Statistical analysis was performed using GraphPad Prism version 9.0.0 (GraphPad Software, CA, USA). The normality of the data were tested using the D'Agostino-Pearson omnibus normality test. We used the Mann–Whitney U test for the comparison of the residual hematoma volume between the two experimental groups. Rotarod results were evaluated using a mixed-effects analysis followed by the Sidák’s post-hoc test. The log-rank (Mantel–Cox) test was used for survival analysis. The observed differences were considered significant when p < 0.05.

## Results & discussion

**Figure 2.**
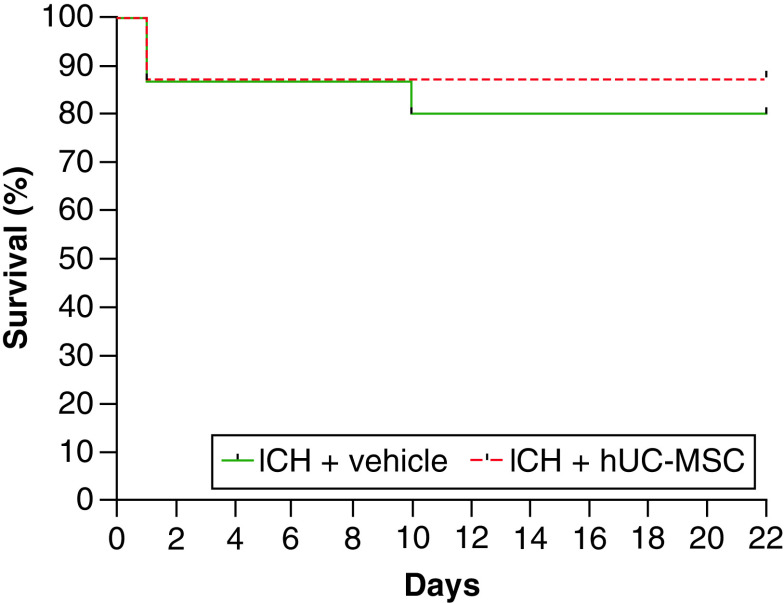
Survival analysis. Animals were treated with (ICH + hUC-MSC) or with the vehicle (ICH + vehicle) 1 h after the induction of ICH. The graph shows the survival curves during the 22 days of follow-up. Log-rank test (Mantel–Cox test) was used. n = 15 (ICH + vehicle) and n = 16 (ICH + hUC-MSC) rats per group. hUC-MSC: Human umbilical cord-derived mesenchymal stromal cells; ICH: Intracerebral hemorrhage.

One animal from the ICH + hUC-MSC group died immediately after the hUC-MSC injection, a potential, but uncommon, complication of the iv. injection of MSC in rodents that had been reported before [15,16]. The remaining animals were followed up for a period of 22 days, during which three deaths occurred in the ICH + vehicle group and one death was registered in the ICH + hUC-MSC group. The survival curves (which include the animal that died immediately after the hUC-MSC injection) are shown in Figure 2. The log-rank (Mantel–Cox) test was used to compare the curves, revealing no statistically significant differences between the groups (χ^2^[1] = 0.2959, p = 0.5865).

**Figure 3.**
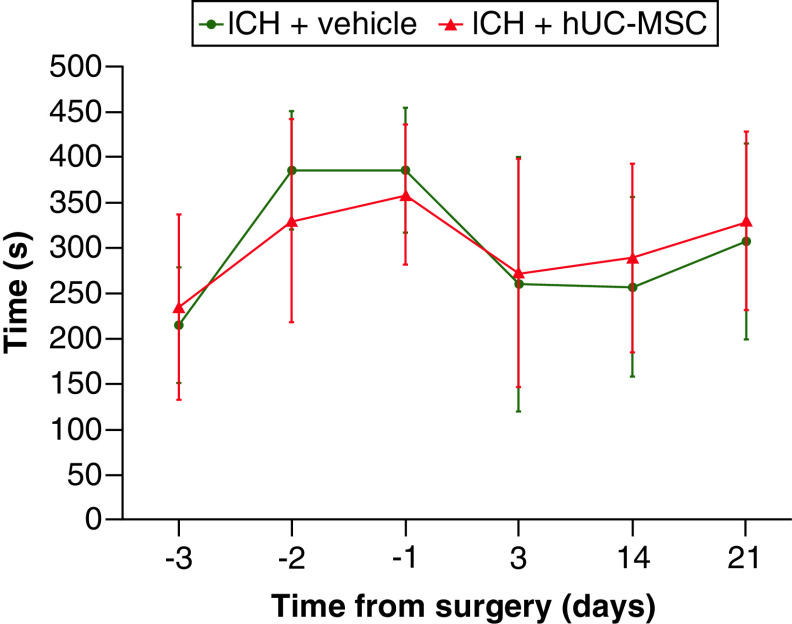
Motor function assessment. Animals were treated with (ICH + hUC-MSC) or with the vehicle (ICH + vehicle) 1 h after the induction of ICH. The graph shows the latencies to fall in the rotarod test at different time points from surgery. Mixed-effects analysis and Sidák’s post-hoc test were used. Data shown in the graph are means ± SD; n = 12-13 animals in the ICH + vehicle group, n = 14 animals in the ICH + hUC-MSC group. hUC-MSC: Human umbilical cord-derived mesenchymal stromal cells; ICH: Intracerebral hemorrhage; SD: Standard deviation.

The rotarod test was employed to assess motor function. Animals were pretested by an examiner blinded to treatment allocation for three consecutive days before ICH, and then tested on days 3, 14 and 21 after ICH (Figure 1). We observed that the performance improved over time during the pre-ICH sessions, followed by an acute worsening on the 3rd day after ICH and a gradual recovery until the last assessment, but no differences were observed between the groups (Figure 3). A mixed-effects analysis revealed an effect of the factor ‘time’ (p < 0.0001) on motor performance, but not of the factor ‘treatment’ (p = 0.9910) and there was no interaction between the factors (p = 0.2758). Intergroup comparisons with the Sidák’s multiple comparison post-hoc test showed no statistically significant differences between the groups at any time point (p ≥ 0.5605). These results indicate that an early treatment with 3  ×  10^6^ hUC-MSC was not able to improve motor function.

**Figure 4.**
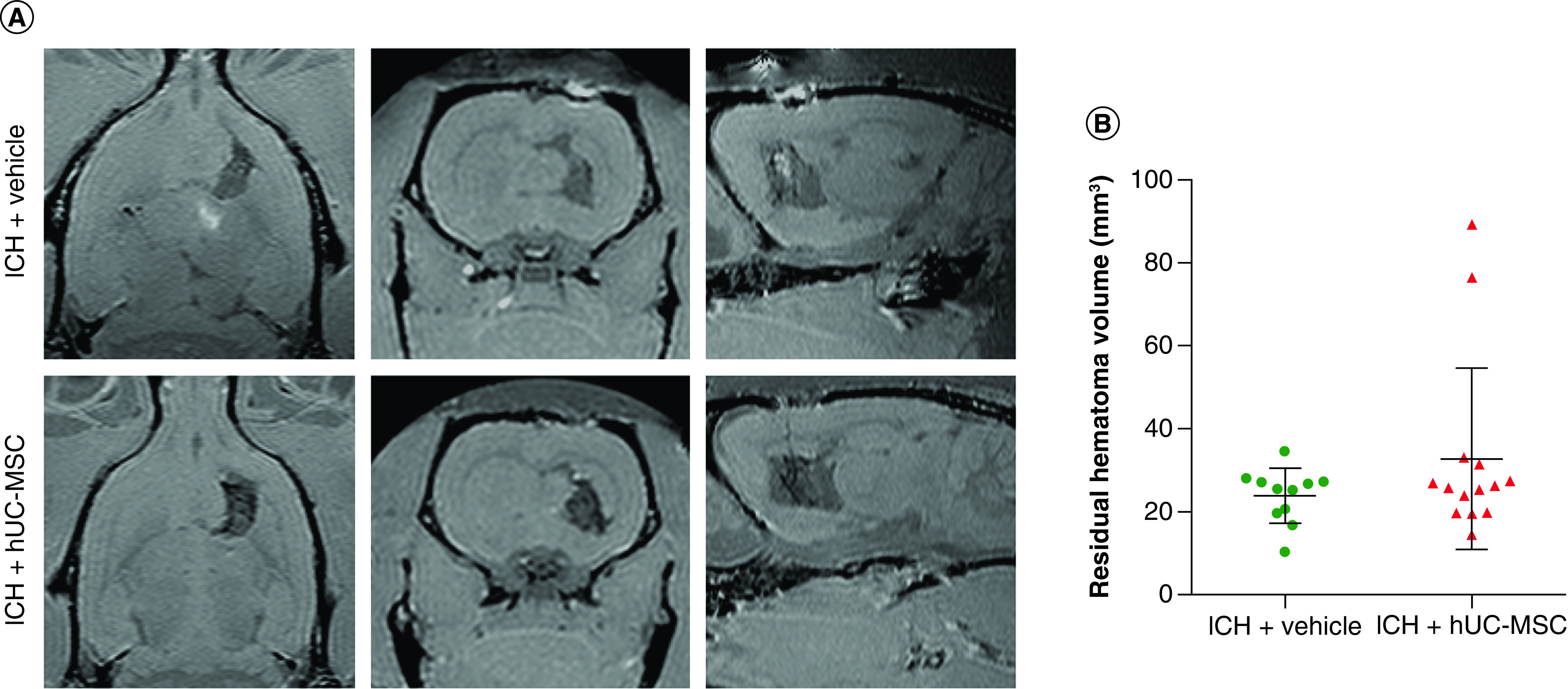
Evaluation of the residual hematoma volume. Animals were treated with (ICH + hUC-MSC) or with the vehicle (ICH + vehicle) 1 h after the induction of ICH. **(A)** Representative T1-weighted images obtained with a 3D sequence in a 2 Tesla MRI scanner 22 days after the induction of ICH. **(B)** Graph showing the quantification of the residual hematoma volume. Data shown in the graphs are individual values, and means ± SD Mann–Whitney U test; n = 11 (ICH + vehicle) and n = 14 (ICH + hUC-MSC) rats per group. hUC-MSC: Human umbilical cord-derived mesenchymal stromal cells; ICH: Intracerebral hemorrhage.

MRI was used to measure the volume of the residual hematoma, which was similar between the groups (Figure 4; U = 66; p = 0.5719; Mann–Whitney U test). The ROUTE method identified two outliers in the ICH + hUC-MSC group, but their removal did not change this result (U = 66; p > 0.9999; Mann–Whitney U test). We cannot rule out the hypothesis that the unexpectedly high residual hematoma volume found in the two outliers from the ICH + hUC-MSC group could be explained by a possible side effect of MSC on hematoma expansion, and this will need attention in future preclinical studies targeting the hyperacute phase of ICH. However, this is unlikely because hUC-MSC have been shown to have a procoagulant activity [17].

Treatments using hUC-MSC offer the possibility of targeting several early pathophysiological processes of ICH, and consequently improving neurological outcomes. In particular, we highlight the capacity of MSC to increase the expression of scavenger receptors and the phagocytic ability of microglial cells and macrophages, which could contribute to the reduction of the residual hematoma volume [18,19]. The immunomodulatory and proregenerative abilities of MSC could facilitate and/or promote neurovascular repair and regeneration [20], and further beneficial effects could also be achieved through the neuroprotective, anti-inflammatory and antioxidant actions attributed to MSC [21–23]. In the present study, however, we found that the iv. injection of 3  ×  10^6^ hUC-MSC 1 h after a severe ICH was not able to improve the performance of rats on the rotarod. Animals from both groups improved their performance similarly over time, which can be attributed to spontaneous functional recovery and/or behavioral compensation [24]. This suggests that additional testing with sensorimotor tests less affected by behavioral compensation, as well as cognitive tests, will be necessary for future studies using this model of severe ICH. In addition, we showed that the residual hematoma volume was not modified by the cell therapy. These results indicate that a single hyperacute injection of hUC-MSC may not be suitable for the treatment of severe ICH.

Although the dose of hUC-MSC that was used in this study has already been shown to be therapeutic in cases of moderate ICH [11], we cannot rule out the possibility that higher doses or different administration strategies (multiple doses or different routes) could be beneficial for severe ICH. The iv. route was chosen in this study for being less invasive and also because the neuroprotective and immunomodulatory effects of MSC are at least partially explained by their systemic actions [7]. In contrast, the administration of MSC into the CNS may allow a greater interaction of MSC with neural cells, which could have therapeutic potential through different mechanisms, but these procedures are associated with possible risks and complications that need to be considered when designing a study [16].

It can also be argued that the transplantation of human cells in immunocompetent animals may not be ideal. However, this is unlikely to be the explanation for the neutral results presented here. MSC are considered to have a lower immunogenic potential than other cell types [25] and the xenogeneic transplantation of human MSC to immunocompetent rats and mice has been extensively tested [7,9,10]. This is also corroborated by our previous findings, as we have shown that hUC-MSC can reduce the residual hematoma volume when administered 24 h after a moderate ICH in immunocompetent rats [11]. In addition, Satani *et al.* [9] have shown in a recent meta-analysis that the improvement of functional outcomes following the administration of bone marrow-derived MSC in ischemic stroke models occurred regardless of the species of the donor and that the iv. route resulted in more improvement in motor function than the intracranial route although similar data are not yet available for ICH.

In this study, the migration of cells to the brain was not evaluated. However, the neutral results reported here are probably not related to the number of cells that were able to enter the brain. We and others have already shown that the number of iv. administered MSC that reach the brain is very low and insufficient to explain their therapeutic effects [11,26,27]. Although the mechanisms of action of MSC are not fully understood, it has been shown that MSC can release extracellular vesicles that could reach injured tissues, and the anti-inflammatory effects of MSC in the spleen are widely recognized [21,23,28]. Recent studies have also found that the majority of iv. injected MSC die after infusion. The phagocytosis and clearance of dying/dead MSC could reprogram monocytes and macrophages, and consequently modulate the different cells with which these phagocytes interact [29,30].

Among the limitations of our study, we acknowledge the lack of a priori sample size determination and the utilization of animals of only one sex (males). However, we have used a slightly larger sample size (n = 11–14) than in our previous study (n = 9–10) [11], in which we detected a significantly lower hematoma volume (around 27% lower) in the group treated with hUC-MSC after a moderate ICH. Studies from other groups have also reported a similar reduction of the hematoma volume ranging from 23.73 [31] to 31.17% [32] after the iv. transplantation of MSC in rats, with sample sizes even smaller than ours (n = 5–8). Moreover, the means were very similar between the groups, which also suggests that a type II error due to insufficient power is unlikely.

## Conclusion

Taken together, our current and previous data [11] indicate that the iv. injection of hUC-MSC in the hyperacute/subacute phases does not seem to be a promising therapeutic approach for severe cases of ICH. Our findings suggest that future clinical trials testing MSC-based therapies in patients with hemorrhagic stroke need to consider the hematoma volume, one of the most important predictors of ICH outcomes [33], for patient selection or stratification. Moreover, the combination of hUC-MSC-based therapies with pharmacological or surgical treatments aimed at restricting hematoma expansion and reducing the toxicity of blood products are strategies that deserve to be investigated in the future.

**Summary points**

- Intracerebral hemorrhage (ICH) is a common cause of neurological morbidity.
- Mesenchymal stromal cells (MSC)-based therapies have been studied as a promising alternative for treating ICH due to their potential to modify multiple pathways associated with brain damage and neurological recovery.
- We have previously shown that a single iv. injection of human umbilical cord Wharton’s jelly-derived MSC (hUC-MSC) 24 h after the induction of ICH reduced the residual hematoma volume in a model of moderate ICH but was inefficient when a severe ICH was modeled in rats.
- Considering that ICH is a medical emergency with rapidly progressing symptoms, we hypothesized that an earlier treatment could be more efficient in severe ICH.
- Here, we show that the hyperacute administration of hUC-MSC did not change the survival rate in a model of collagenase-induced severe ICH in rats.
- Motor function was not improved by the hyperacute hUC-MSC treatment.
- Hyperacute hUC-MSC transplantation did not altered the residual hematoma volume after a severe ICH.
- These results indicate the lack of efficacy of the iv. administration of hUC-MSC in the hyperacute phase of severe ICH.
